# Eruptive Pruritic Papular Porokeratosis (EPPP) Presenting as a Rare Facial Manifestation Associated With COVID-19: A Case Report

**DOI:** 10.7759/cureus.57650

**Published:** 2024-04-05

**Authors:** Sabita Aryal, Zhuqian Jiang, Liu Ye Qiang, Abdullah Shehryar

**Affiliations:** 1 Dermatology and Venerology, Shanghai Skin Disease Hospital, Tongji University, Shanghai, CHN; 2 Dermatology, Shanghai Jiading Hospital of Traditional Chinese Medicine, Shanghai, CHN; 3 Dermatology, Shanghai Skin Disease Hospital, Tongji University, Shanghai, CHN; 4 Internal Medicine, Allama Iqbal Medical College, Lahore, PAK

**Keywords:** case report, inflammatory dsp, epidermal keratinocytes, covid-19, eruptive pruritic papular porokeratosis (eppp), porokeratosis

## Abstract

This case report presents a rare instance of Eruptive Pruritic Papular Porokeratosis (EPPP) in a 71-year-old Chinese male, emerging on atypical sites (face, scalp, and ears) following a COVID-19 infection, and explores the potential link between viral infections and EPPP onset. The patient's lesions, characterized by annular brown patches with hyperkeratotic ridges, showed significant improvement following treatment with Baricitinib and Acitretin. This case underscores the need for awareness of unusual presentations of EPPP and suggests the potential efficacy of Janus Kinase (JAK) inhibitors in treatment, prompting further research into the pathophysiological connections between EPPP and viral infections. Adherence to the SCARE 2023 guidelines ensures a comprehensive and transparent case presentation.

## Introduction

Eruptive Pruritic Papular Porokeratosis (EPPP) is an unusual variant of Disseminated Superficial Porokeratosis (DSP), a subtype of porokeratosis clinically characterized by grouped, abrupt, eruptive, and intensely pruritic skin lesions distributed over the trunk and extremities [1-2]. Of the several ways that porokeratosis presents itself, EPPP stands out as a particularly difficult case because of its abrupt onset, widespread distribution, and severe itchy symptoms. The usual course of the condition is months or years of asymptomatic DSP, followed by acute pruritic exacerbations, which eventually go away on their own within a year [2]. Histologically, EPPP shows the features of DSP. Although secondary amyloid deposition is occasionally seen in porokeratosis, its association with EPPP is extremely rare [3]. The precise causes and correlations of EPPP remain unclear, even though the broad traits of porokeratosis subtypes are well-studied. Contemporary research has looked into possible connections between viral infections and the development of EPPP, providing new opportunities to study how the immune system functions in the pathophysiology of this illness. Research on the complex interactions among viral triggers, inflammatory responses, and neoplastic keratinocyte clones is continuing. This case report provides a novel viewpoint to the body of literature by describing a case of EPPP presented at atypical facial regions after a confirmed COVID-19 episode. We include details, particularly on clinical information, histopathology results, and therapeutic approaches, to enhance the developing narrative of EPPP. Furthermore, in accordance with the SCARE 2023 guidelines, this case report has been structured to meet the recommended criteria for transparent and comprehensive reporting of surgical and case studies [4].

## Case presentation

A 71-year-old Chinese male from a socio-economically disadvantaged background presented with a one-month history of a rash on the face with greasy crusts in the hospital. The rash manifested after confirmed COVID-19 infections, as attested by the patient's results during the mandatory mass PCR testing conducted by the Shanghai City Government in China. During this period, he experienced symptoms consistent with the viral infections, self-managed via analgesics for symptomatic relief, and did not warrant hospitalization. On the 13th day of illness, a PCR test yielded negative results. Soon after recovering from COVID-19, the patient developed a rash on his face approximately 2-3 weeks later.

According to the dermatologist physician team of the hospital, the patient had no reported history of hypertension, coronary heart disease, diabetes mellitus, fatty liver, obesity, or family history of porokeratosis and was not actively using any medications, or with any comorbidities. Comprehensive investigations were conducted to assess the patient's condition. The complete blood count (CBC) revealed normal levels of red blood cells, white blood cells, and platelets, indicating no significant abnormalities. Liver function tests (LFT) showed normal levels of enzymes, proteins, and bilirubin, indicating healthy liver function. Renal function tests (RFT) indicated normal kidney function, with creatinine and blood urea nitrogen (BUN) within the normal range. Viral serology tests for HIV, HCV, and HBsAg were negative. However, certain additional investigations, such as peripheral blood smears and serum immunoglobulin tests, were not performed due to the patient's denial and socioeconomic constraints.

**Figure 1:**
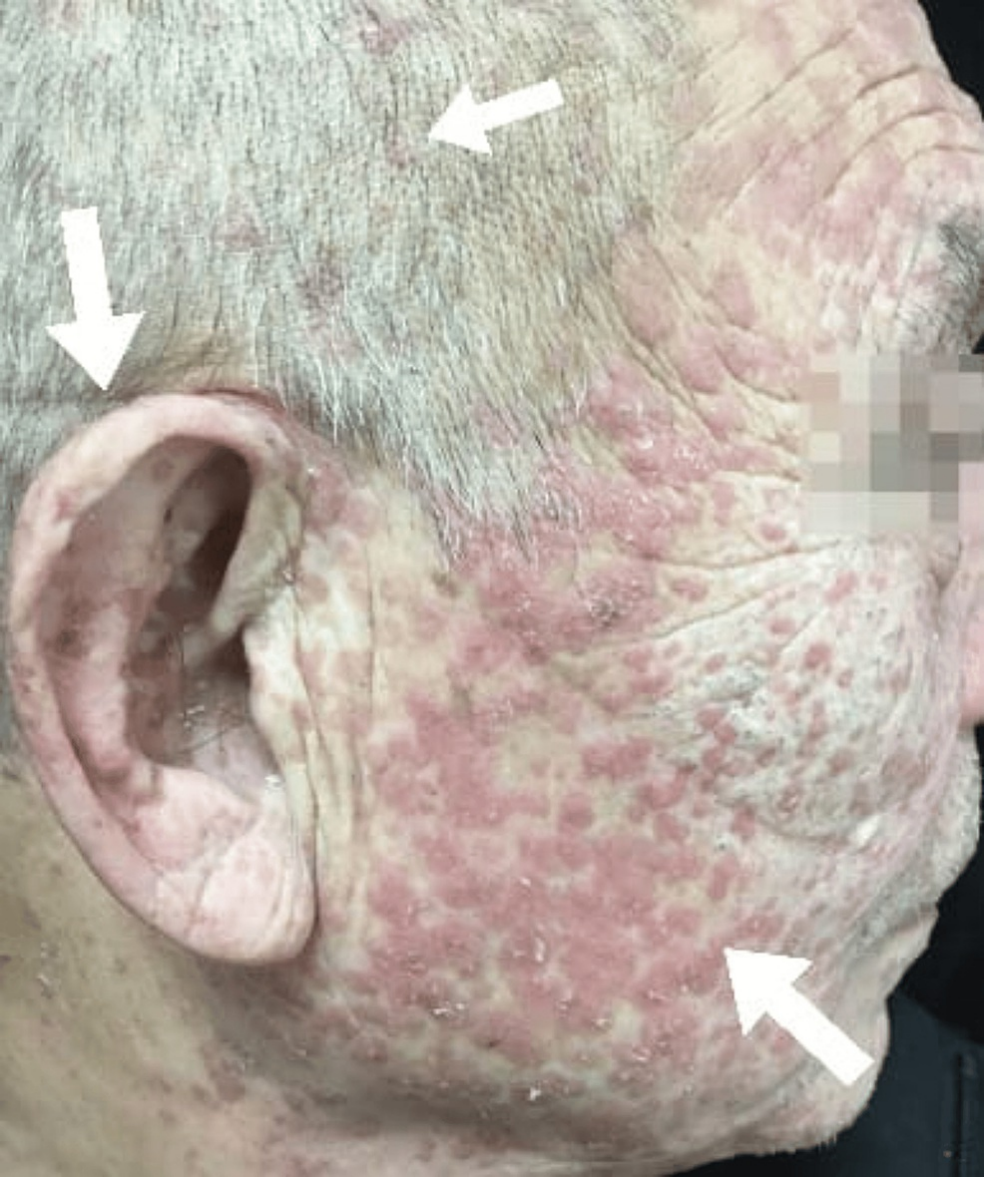
Multiple intensely pruritic macules and annular erythematous papules on the face, ears and scalp. Lesions are illustrated by the arrows.

Physical examinations revealed multiple annular brown lesions measuring up to 10 mm in diameter, surrounded by raised hyperkeratotic ridges on the face extended to the scalp and neck. His palms, soles, and oral mucosa were spared, but bilateral auricles were involved. Dermoscopy found an annular hyperkeratotic track and rim with a double edge, leaving behind small brown spots and annular lesions. All of the lesions on the face, scalp and ear are illustrated in Figure 1.

**Figure 2:**
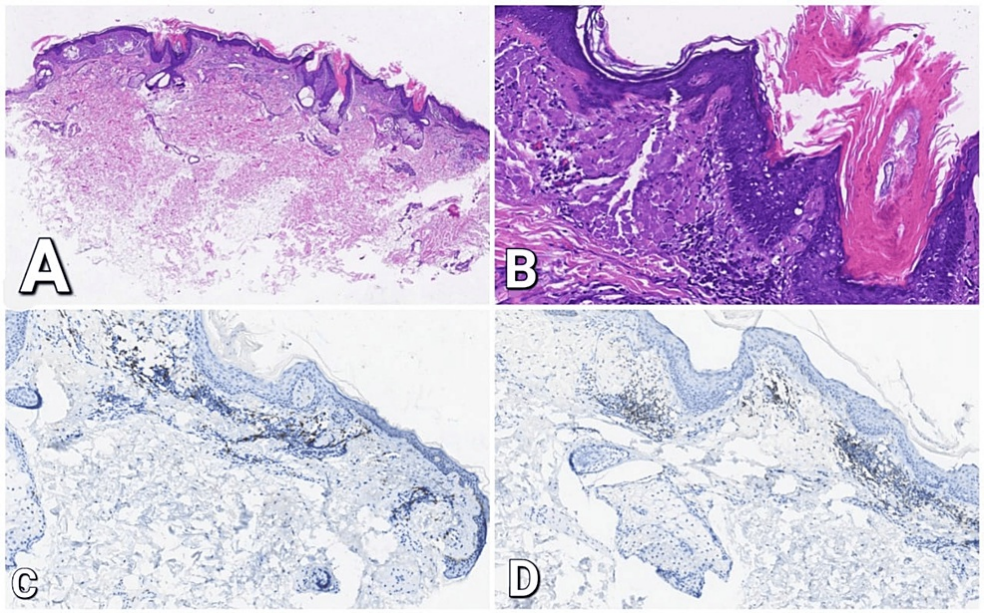
Histopathology revealing cylindrical parakeratosis with dense superficial perivascular mononuclear inflammatory infiltrate (B) in the dermis along with amyloid depositions (A); immunohistochemical staining shows the presence of CD4+ helper T-cells (C) intermingled CD8+ suppressor T-Cells (D).

The histopathology exam showed a scattered red mass revealing cylindrical parakeratosis with perivascular lymphoid inflammatory infiltrates (Figure 2A) and amorphous substance deposition in the cornoid lamella (Figure 2B). Immunohistochemical staining identified CD4+ helper T-cells as predominant in dermal infiltrate (Figure 2C), with intermingled CD8+ suppressor/cytotoxic T cells (Figure 2D).

The patient was diagnosed with EPPP and was recommended Baricitinib 2 mg, a selective Janus Kinase (JAK) inhibitor, administered orally once daily for two weeks. In addition, the patient was prescribed an oral retinoid medication, Acitretin capsules (10 mg), orally once daily for 30 days. Following the medication administration, we observed a high treatment efficacy without any reported side effects.

**Figure 3:**
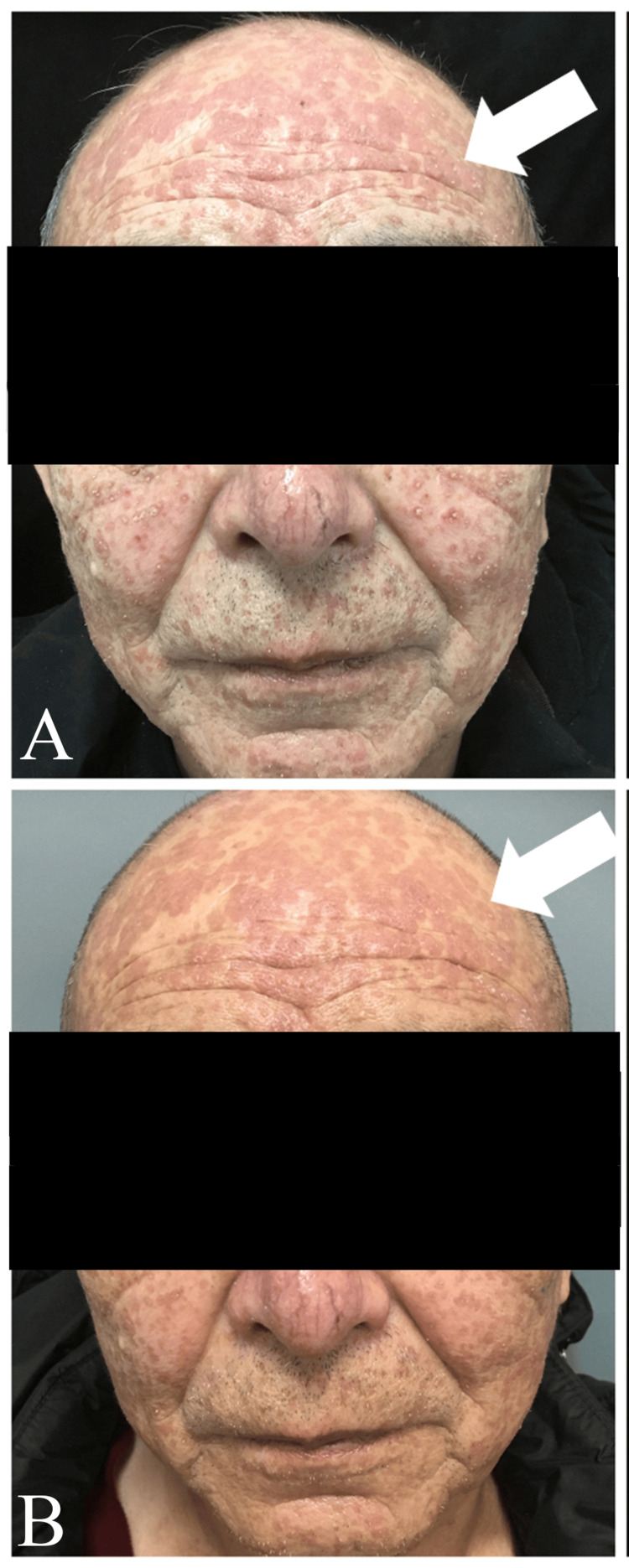
Follow-up clinical gross image and dermoscopy examination images of the lesions after 15 days of treatment (A) and after 30 days of treatment (B). Areas of interest are marked by the arrows.

Baricitinib, a JAK inhibitor primarily prescribed for rheumatoid arthritis, demonstrated effectiveness in this case despite its limited utilization in cutaneous conditions. The patient responded well, reporting improved comfort after two weeks of therapy. Follow-up assessments were conducted 15 days and one month after the initial diagnosis; however, due to socioeconomic constraints, the patient did not adhere to regular follow-up visits. Notably, the patient exhibited significant improvement, with visible regression in lesions (Figure 3). The patient has reported significant improvement and a return to normal daily activities during follow-up conducted via telephone consultations.

## Discussion

EPPP is a relapsing and rare variant of DSP. It is defined as having a rapid onset (<2 months), disseminated lesions (>100 lesions involving multiple regions), and histopathology consistent with porokeratosis [5]. EPPP, including other types of inflammatory porokeratosis, is more commonly seen in older individuals, with males being affected twice as often as females [6]. The lesions preferably appear on the trunk, arms, and legs [5]. In contrast, our patient presented with uncommon EPPP on the face, scalp, and ears.

The underlying cause of EPPP remains unclear and requires further investigation. It is suggested that immunological reactions against neoplastic keratinocyte clones in porokeratosis may lead to inflammation in patients with inflammatory DSP [7]. This implies that there could be a progression from DSP to EPPP, possibly due to some underlying causes, but it is still unclear.

Additionally, the etiology of EPPP may be attributed to viral infections, immunosuppression, and transplantation in some cases [8]. In the literature, out of 32 examples of EPPP, six individuals had a viral infection: hepatitis C virus (4/6), hepatitis B virus (1/6), and recurrent herpes simplex virus (1/6) [9]. While the association between systemic immunosuppression and porokeratosis is well recognized, the precise relationship between immunosuppression and the emergence of porokeratosis is still not fully understood [10]. One theory suggests that immunosuppression may directly or indirectly increase the population of epidermal keratinocytes [10]. The inflammatory response observed in EPPP may be attributed to an immunological reaction against tumorigenic keratinocyte clones, leading to alterations in keratinocyte maturation or increased epidermal proliferation [11].

In response to the treatment with Baricitinib and Acitretin for EPPP, the patient demonstrated significant clinical improvement, quantitatively supported by reductions in the Visual Analog Scale (VAS) score for itch from 8 to 2, indicating a substantial decrease in itch severity. Furthermore, the 12-item pruritus severity scale (12-PSS) score decreased from 45 to 10, reflecting an improvement in pruritus severity and its impact on daily life. Lastly, the Dermatology Life Quality Index (DLQI) score improved from 24 to 5, showcasing a marked enhancement in the patient's quality of life post-treatment. These improvements highlight the therapeutic efficacy of the treatment regimen, highlighting its potential utility in managing cutaneous conditions like EPPP, even in the absence of extensive prior use in similar contexts.

It has been reported that cases of severe COVID-19 infection exhibit lymphocyte depletion, suggesting immunosuppression [12]. Studies have reported erythematous and pruritic skin lesions associated with COVID-19, supporting a potential link between the infection and the patient's rash. Maculopapular eruptions (47%) have been observed in a significant number of confirmed or suspected COVID-19 cases (375) in a nationwide rapid study in Spain [13]. Although our patient's rash was not associated with severe illness, we should consider the possibility that moderate COVID-19 cases were underrepresented in that study, which may explain the disparity. Nevertheless, the presence of COVID-19 in our patient suggests a potential link to the development of the rash.

Histologically, EPPP papular lesions usually have dense superficial perivascular mononuclear inflammatory infiltrates with eosinophils [14,15]. Reed and Leone suggested localized keratinocytic clones cause epidermal dysplasia [16]. Secondary amyloid deposition is rare in EPPP, but positive staining for 34BE12 suggests that amyloid materials originate from degenerating epidermal keratinocytes [4]. Skin biopsies from our patient showed colloid bodies along with a rare observation of amyloid deposits.

CD8+ lymphocytes found in EPPP may target abnormal keratinocyte clones in EPPP, distinguishing them from the CD4+ cell-mediated response in DSP [2, 15]. Furthermore, Tanaka et al. found CD8+ and CD1a+ Langerhans cells and CD4+ T cells in the dermis of pruritic inflammatory lesions and hypothesized that a T-cell-mediated immune response against abnormal epidermal clones is important for the regression of multiple porokeratosis lesions [17].

Despite the presence of both CD4+ and CD8+ T-cells in the dermal infiltrate of our patient, there was no observed regression of the lesions. This T-cell profile suggests an immune imbalance, where CD4+ helper T-cells are responsible for coordinating immune responses, while CD8+ suppressor/cytotoxic T-cells play a role in regulating immune activity. The lack of lesion regression indicates that other factors may be contributing to the pathogenesis and progression of the disease. One noteworthy feature of our case is that JAK inhibitors are used to treat EPPP. Our patient showed considerable improvement in comfort and lesion reduction as a result of its efficacy. Baricitinib is a reversible Janus-associated kinase (JAK) inhibitor that blocks the signaling of several cytokines linked to COVID-19 immunopathology. By focusing on host factors that viruses depend on for cell entry and by inhibiting type I interferon-driven angiotensin-converting enzyme-2 overexpression, it may also have antiviral effects [18]. In EPPP, there is evidence of eosinophil and lymphocyte infiltration, which advances the skin injury. A recent study by Xia and Jiang suggests that the existence of eosinophilic activity and lymphocytic inflammation indicates a possible role for immunomodulation in the management of EPPP. This study supports the effective use of a JAK inhibitor, which is well-known for its anti-inflammatory qualities, in the treatment of EPPP [19].

Additionally, it's worth noting that no standard treatment for EPPP exists. Options include topical 5-FU, steroids, imiquimod, cryotherapy, carbon dioxide laser [20], or a frequency-doubled Q-switched Nd: YAG laser can be considered if the lesions cause other problems or are cosmetically unacceptable. Pruritic symptoms may resolve on their own in a few months in most cases, but antihistamines can relieve discomfort [21].

The study had certain limitations that should be acknowledged. Firstly, due to the strict lockdown measures during the study period, the patient was unable to undergo prompt confirmation tests for COVID-19 infections and viral antibody status at the hospital premises, resulting in delayed diagnostic information. Additionally, the patient's socioeconomic circumstances and limited resources hindered comprehensive data collection and thorough assessments, as multiple hospital visits for follow-up were not feasible. These limitations highlight the challenges faced in gathering detailed information and conducting extensive follow-up. Furthermore, as this case report includes only one patient, the findings may not fully represent the diversity of experiences and outcomes in other individuals with EPPP. The absence of a control group also limits the ability to compare the effectiveness of the treatment approach used. Moreover, the lack of long-term follow-up restricts our understanding of treatment efficacy and potential side effects over an extended period.

## Conclusions

This case enhances our comprehension of Eruptive Pruritic Papular Porokeratosis (EPPP) by shedding light on its potential association with viral infections and its distinctive manifestation on the face and ears. It emphasizes the importance of considering atypical locations when diagnosing this rare dermatological condition. The potential link between COVID-19 and EPPP onset raises intriguing questions about viral infections' role in porokeratosis variants, warranting further mechanistic exploration. Moreover, the presence of secondary amyloid deposition in EPPP, although rare, expands our understanding of histopathological features in the disease and necessitates a more in-depth investigation into its implications for disease progression and management.

## References

[1] Kanekura T, Yoshii N (2006). Eruptive pruritic papular porokeratosis: a pruritic variant of porokeratosis. J Dermatol.

[2] Tee SI, Chong WS (2012). Eruptive pruritic papular porokeratosis. Indian J Dermatol Venereol Leprol.

[3] Yamamoto T, Furukawa H, Ohtsuka M (2013). Amyloid deposition in disseminated superficial porokeratosis with inflammatory stages. J Dermatol.

[4] Sohrabi C, Mathew G, Maria N, Kerwan A, Franchi T, Agha RA (2023). The SCARE 2023 guideline: updating consensus Surgical CAse REport (SCARE) guidelines. Int J Surg.

[5] Wu CY, Chiu HC, Jee SH (2019). Eruptive disseminated porokeratosis: a rare variant and therapeutic intervention. Dermatologica Sinica.

[6] Shoimer I, Robertson LH, Storwick G, Haber RM (2014). Eruptive disseminated porokeratosis: a new classification system. J Am Acad Dermatol.

[7] Wakatabi K, Kakurai M, Yamada T, Umemoto N, Demitsu T, Yoneda K (2012). Inflammatory disseminated superficial porokeratosis with an unusual clinical feature of the pruritic, erythematous papules preceding annular brownish pigmentation. J Dermatol.

[8] Schena D, Papagrigoraki A, Frigo A, Girolomoni G (2010). Eruptive disseminated porokeratosis associated with internal malignancies: a case report. Cutis.

[9] Vargas-Mora P, Morgado-Carrasco D, Fustà-Novell X (2020). Porokeratosis: a review of its pathophysiology, clinical manifestations, diagnosis, and treatment [Article in English, Spanish]. Actas Dermosifiliogr (Engl Ed).

[10] Bednarek R, Ezra N, Toubin Y, Linos K, Mousdicas N (2015). Eruptive disseminated porokeratosis associated with corticosteroid-induced immunosuppression. Clin Exp Dermatol.

[11] Morgado-Carrasco D, Feola H, Fustà-Novell X (2020). Eruptive pruritic papular porokeratosis or inflammatory form of disseminated superficial porokeratosis: a new case and review of the literature. Dermatol Online J.

[12] Phetsouphanh C, Darley DR, Wilson DB (2022). Immunological dysfunction persists for 8 months following initial mild-to-moderate SARS-CoV-2 infection. Nat Immunol.

[13] Galván Casas C, Català A, Carretero Hernández G (2020). Classification of the cutaneous manifestations of COVID-19: a rapid prospective nationwide consensus study in Spain with 375 cases. Br J Dermatol.

[14] Biswas A (2015). Cornoid lamellation revisited: apropos of porokeratosis with emphasis on unusual clinicopathological variants. Am J Dermatopathol.

[15] Luo LL, Chen H, Zeng XS, Cui PG (2021). A case report of inflammatory disseminated superficial porokeratosis: an eruptive pruritic papular variant of porokeratosis. Int J Dermatol Venereol.

[16] Reed RJ, Leone P (1970). Porokeratosis—A mutant clonal keratosis of the epidermis: I. Histogenesis. Arch Dermatol.

[17] Tanaka M, Terui T, Kudo K, Tagami H (1995). Inflammatory disseminated superficial porokeratosis followed by regression. Br J Dermatol.

[18] Jorgensen SC, Tse CL, Burry L, Dresser LD (2020). Baricitinib: a review of pharmacology, safety, and emerging clinical experience in COVID-19. Pharmacotherapy.

[19] Xia J, Jiang G (2023). A report of eruptive pruritic papular porokeratosis treated with abrocitinib. Clin Cosmet Investig Dermatol.

[20] Stork J, Kodetová D (1997). Disseminated superficial porokeratosis: an eruptive pruritic papular variant. Dermatology.

[21] Mcdonald SG, Peterka ES (1983). Porokeratosis (Mibelli): treatment with topical 5-fluorouracil. J Am Acad Dermatol.

